# Ultrasensitive optical reflectivity in annular nanohole array on Photonic crystal slab based on bound states in the continuum

**DOI:** 10.1038/s41598-018-29930-5

**Published:** 2018-08-20

**Authors:** Zhijian Li, Qing Zhu, Yaonan Wang, Suxia Xie

**Affiliations:** 1 College of Electrical and Information Engineering, Hunan University, Changsha, P.R. China; 2 National Engineering Laboratory for Robot Visual Perception and Control Technology, Hunan University, Changsha, P.R. China; 3 Department of Electrical and Computer Engineering, University of Wisconsin, Madison, WI USA; 4 School of Physics and Electronic Science, Hunan University of Science and Technology, Xiangtan, P.R. China

## Abstract

We investigate optical bound states in the continuum (BICs) supported by a photonic crystal (PhC) slab penetrated with periodic annular holes theoretically. Ultrahigh-quality factor (Q-factor) resonances associated with BICs are obtained with a Q-factor more than 10^8^. The BICs can be seen at nonzero incident angles by tuning the lattice constant, layer thickness, inner pillar radius and the refractive index of the surrounding medium, and figure of merit (FOM) at the BICs can reach infinite theoretically. New Fano resonance line appears with BICs when the annular hole’s symmetry is broken, which can be attributed to the change of the waveguide modes and their coupling when the annular hole shape is asymmetrical. We confirm it by tuning the inner pillars’ location and size to realize the structure’s asymmetry. It is shown the location and size asymmetry of the inner pillars inside each outer hole can impact the reflectivity and the formation of the BICs obviously. Results from finite difference time domain method (FDTD) simulation and temporal coupled mode theory (CMT) calculations agree well, which are beneficial to design elements based on optical BICs in various applications, such as biosensors, perfect filters, and waveguides.

## Introduction

Quality factor (Q-factor) controlling of nanostructures is fundamentally important in electromagnetism. There are potential applications of devices with high Q-factor such as spanning oscillators, filters, antennas, sensors, nanolasers, or single-photon sources^1–6^. Q-factor is usually limited by the inherent material properties such as the Ohmic and dielectric and some other factors, for example, radiation losses, which can be controlled efficiently. More recently, Bound states in the continuum (BICs) with infinite Q-factor have attracted many attentions in optics. BICs were proposed by von Neumann and Wigner in 1929^7^. They constructed a 3D potential extending to infinity and oscillating in a way that was tailored to support an electronic BICs mathematically. Their BICs supporting system is too arbitrary to be realized. But this proposal leads to BICs widely identified in different systems with different mechanisms theoretically and experimentally. For example, electronic BICs have been predicted in atomic and molecular systems^8,9^ and in artificial systems later^10,11^. Investigations of BICs in optics spring up recently. The term BICs was first represented in optics around 2008^12,13^, and experimental observation of optical BICs followed only in 2011^14^. In 2013, Chia Wei Hsu *et al.*^15^ observed the trapped light within the radiation continuum experimentally using PhC slab with hole array, and the first BIC laser is realized in 2017 by Ashok Kodigala *et al.*^6^. In photonics, BICs have been shown to exist in dielectric gratings^12^, photonic crystal cavities^13^, lossless core-shell particles^16^, coupled waveguide arrays^17–20^, and photonic crystal slabs^15^. BICs have robustness against changing of system parameters, which makes it very promising for many applications ranging from on-chip photonics and optical communications^6,17,21^ to biological sensing^22^ and photovoltaics.

In this paper, we investigate optical bound states in the continuum (BICs) supported by a photonic crystal (PhC) slab penetrated with periodic annular holes theoretically. Ultrahigh-quality factor (Q-factor) resonances associated with BICs are obtained with a Q-factor more than 10^8^. The BICs can be seen at nonzero incident angles by tuning the lattice constant, layer thickness, inner pillar radius and the refractive index of the surrounding medium, and figure of merit (FOM) at the BICs can reach infinite theoretically. New Fano resonance line appears with BICs when the annular hole’s symmetry is broken, which may be attributed to the change of the waveguide modes and their coupling when the annular hole shape is asymmetrical. We confirm it by tuning the inner pillars’ location and size to realize the structure’s asymmetry. It is shown the location and size asymmetry of the inner pillars inside each outer hole can impact the reflectivity and the formation of the BICs obviously. In order to prove the validity of the investigation, we compare the finite difference time domain (FDTD) method^23,24^ simulation results with that of the temporal coupled mode theory (CMT)^25^ calculations. It is found that results from both methods agree very well. Results in this paper are beneficial to design practical resonance elements based on optical BICs in various applications, such as biosensors, perfect filters, and waveguides.

## Results and Discussion

**Figure 1:**
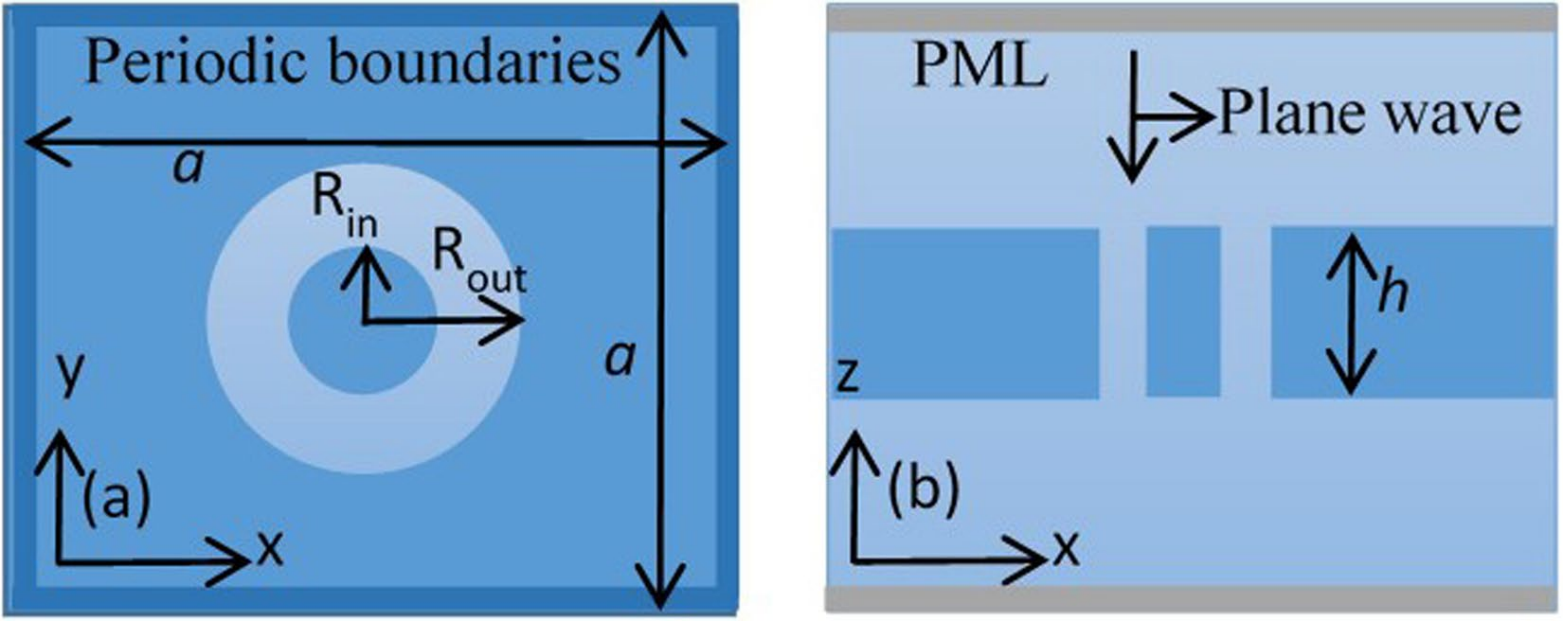
Schematics of Si_3_N_4_ photonic crystal (PhC) slab with a square array of annular cylindrical holes. (**a**) Top view of the slab with cylindrical annular hole shapes in *x*-*y* cross-section, and (**b**) side view of the structure in *x*-*z* cross-section.

We report numerical simulation results of reflectivity based on a schematic of Si_3_N_4_ photonic crystal (PhC) slab with a square array of annular cylindrical holes surrounded by silica medium. The results of these structures are simulated using the FDTD method, compared by analytical results by CMT, in order to show the optical BICs. In the simulation model, perfectly matched layer (PML)^26^ boundary conditions are used at the top and bottom of the lattice in the z-direction. Periodic boundary conditions are used in *x* and *y* directions respectively due to the periodicity of the structure. We send a Gaussian single pulse of light with a wide frequency profile, and it impinges on the structure in -*z*-direction with a polarization along the *y*-direction. Schematics of Si_3_N_4_ photonic crystal (PhC) slab with a square array of annular cylindrical holes are shown in Fig. 1. Figure 1(a) is the top view of the slab with cylindrical annular hole shapes in the *x*-*y* cross-section and Fig. 1(b) is the side view of the structure in the *x*-*z* cross-section.

We consider Si_3_N_4_ PhC slab with a refractive index 2.02. The PhC slab is immersed in an optical medium with a refractive index *n*. Lattice constant, slab thickness, inner and outer radii of the hole are denoted as *a*, *h*, *R*_*in*_, *R*_*out*_, respectively.

**Figure 2:**
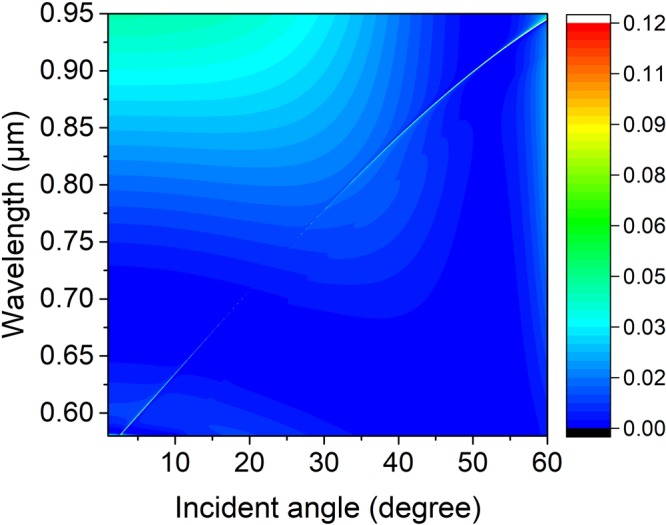
Numerical reflectivity spectra for the p-polarized wave as a function of incident angle *θ* and wavelength *λ* of the periodic symmetrical annular hole array on a PhC Si_3_N_4_ slab. Where the slab thickness, annular hole’s inner and outer radii, lattice constant, and surrounding medium refractive index are *h* = 180 nm, *R*_*in*_ = 50 nm, *R*_*out*_ = 100 nm, *a* = 336 nm, and *n* = 1.46, respectively.

Light incident on the PhC slab excites the guided resonance, interferes with the leaky modes, and creates sharp Fano features in the reflectivity spectrum^27^. In order to confirm the existence of the trapped state, an angle-resolved reflectivity simulation map of the structure normalized to the reflectivity spectra of the incidence is simulated by using FDTD in Fig. 2 with parameters of the lattice constant, PhC slab thickness, outer radius, the inner radius and surrounding medium index being denoted as 336 nm, 180 nm, 100 nm, 50 nm, and 1.46, respectively. A resonance shows up in the appearance of a thin faint line extending from the bottom left corner to the top right corner, this resonance disappears near 23 degree, which indicates there exists a trapped state with no leakage. As a result of decoupling from far-field radiation, a perfect bound state has no Fano feature. In the simulation, it is indeed observed that the Fano feature of the p-polarized wave is very faint and disappears near 23 degree as shown in Fig. 2. The interruption of the faint resonance line indicates the region where a BIC occurs with an infinite high Q-factor. The physical origin of the ultrahigh-Q factor resonance is related to simultaneous suppression of the leakage radiation amplitudes to the zero-order waves in upper and under mediums of the PhC layer. This effect involves interference between partial leakage radiations from different Bloch modes and complex interactions of evanescent fields at the top and bottom interfaces of the film^15,28^.

In order to gain deeper insight into the physics of the FDTD simulated resonance, we develop a CMT that accounts for the presence of guided leaky resonances in the Si_3_N_4_ layer. By exciting the model with an incident source propagating from the top and impinging on to the Si_3_N_4_ layer hole, the reflectivity of the structure can be attained from first-order perturbation to Maxwell’s equation, energy conservation considerations, and neglecting second order effects.

In CMT, the field of the resonance and fields *S*_*m*±_ of the incoming/outgoing plane waves are considered separate entities that are weakly coupled to each other through their spatial overlaps^29,30^. The resonance decays with a radiative-decay lifetime *τ*_*r*_ from leakage into the outgoing plane waves, and non-radiative-decay lifetime *τ*_*nr*_ from material absorption and disorder scattering. As we will see, the effect of *τ*_*nr*_ is to broaden the resonance feature in the reflectivity spectrum; therefore it also heuristically accounts for the inhomogeneous broadening in the measured reflectivity data. Incoming plane waves excite the resonance with coupling coefficients denoted by *k*_1_ and *k*_2_. Thus we have

1 $$\frac{dA}{dt}=(-{i{\rm{\omega }}}_{0}-\frac{1}{{\tau }_{r}}-\frac{1}{{\tau }_{nr}})A+{k}_{1}{s}_{1+}+{k}_{2}{s}_{2+}$$

The plane waves on the two sides of the slab couple to each other through a direct scattering process, with transmission and reflectivity coefficients *t*_*slab*_ and *r*_*slab*_. The resonance decays into the outgoing plane waves, with coupling coefficients denoted by *d*_1_ and *d*_2_. Therefore,

2 $${s}_{1-}={r}_{slab}{s}_{1+}+{t}_{slab}{s}_{2+}+{d}_{1}A$$

3 $${s}_{2-}={t}_{slab}{s}_{1+}+{r}_{slab}{s}_{2+}+{d}_{2}A$$

4 $${s}_{2+}=0$$

Now, assume ${e}^{-i\omega t}$ time dependence for the resonance amplitude A. solving Eqs (1–4) as a system of linear equations, we obtain

5 $$\frac{{s}_{1-}}{{s}_{1+}}={r}_{slab}+\frac{{d}_{1}{k}_{1}}{-i\omega +i{\omega }_{0}+\frac{1}{{\tau }_{r}}+\frac{1}{{\tau }_{nr}}}$$

Here we set *γ* = $\frac{1}{{\tau }_{r}}+\frac{1}{{\tau }_{nr}}$, *d*_1_*k*_1_ = *fγ*, the factor *f* is the complex amplitude of the resonant mode, and it can be obtained as *f* = *t*_*slab*_ + *r*_*slab*_.

Then the reflected amplitude *r* can be expressed as follows:

6 $$r=\frac{{s}_{1-}}{{s}_{1+}}={r}_{slab}+f\frac{{\rm{\gamma }}}{-\,i\omega +i{\omega }_{0}+{\rm{\gamma }}}$$

we can write the overall reflectivity as

7 $$R={|r|}^{2}$$

The parameter *r*_*slab*_ and *t*_*slab*_ represent the background of the spectra. These parameters can be determined by fitting the background to the response spectra of a uniform slab, as

8 $${r}_{slab}=\frac{i\frac{{k}_{z0}^{2}-{k}_{z1}^{2}}{2{k}_{z0}{k}_{z1}}\,\sin ({k}_{z1}h)}{\cos ({k}_{z1}h)-i\frac{{k}_{z0}^{2}+{k}_{z1}^{2}}{2{k}_{z0}{k}_{z1}}\,\sin ({k}_{z1}h)}$$

9 $${t}_{slab}=\frac{1}{\cos ({k}_{z1}h)-i\frac{{k}_{z0}^{2}+{k}_{z1}^{2}}{2{k}_{z0}{k}_{z1}}\,\sin ({k}_{z1}h)}$$

for a plane wave with parallel wave vector *k*_*x*_, incident from a dielectric material with a dielectric constant *ε*_0_ = 2.13, through a uniform dielectric slab with a thickness *h* and a dielectric constant *ε*_1_. The parameters *k*_*z0*_ and *k*_*z*1_ in Eqs (8, 9) represent the wave vector components along the z-axis in the uniform slab and are defined as

10 $${k}_{z0}=\sqrt{{\varepsilon }_{0}\frac{{\omega }^{2}}{{c}^{2}}-{k}_{x}^{2}}$$

11 $${k}_{z1}=\sqrt{{\varepsilon }_{1}\frac{{\omega }^{2}}{{c}^{2}}-{k}_{x}^{2}}$$

In obtained Eqs (10, 11), we assume a positive frequency convention, in order to be consistent with the Lorentzian functions that we have chosen for the resonance in Eq. (5).

The dielectric constant of the uniform slab *ε*_1_, as obtained by the fitting procedure, represents an effective dielectric constant for the photonic crystal. Due to the presence of the holes, such *ε*_1_ is a slowly varying function of the frequency. At low frequencies, the wavelength of incident light is large, and *ε*_1_ for this polarization approaches the average dielectric constant of the crystal. At higher frequencies, as the incident wave probes more details of the crystal structure, *ε*_*1*_ starts to deviate from the average dielectric constant. Within the frequency range, we have found that a frequency-dependent dielectric constant

12 $${\varepsilon }_{1}(\omega )=2\pi c/(15\omega )-2$$

gives a good fit for the background.

**Figure 3:**
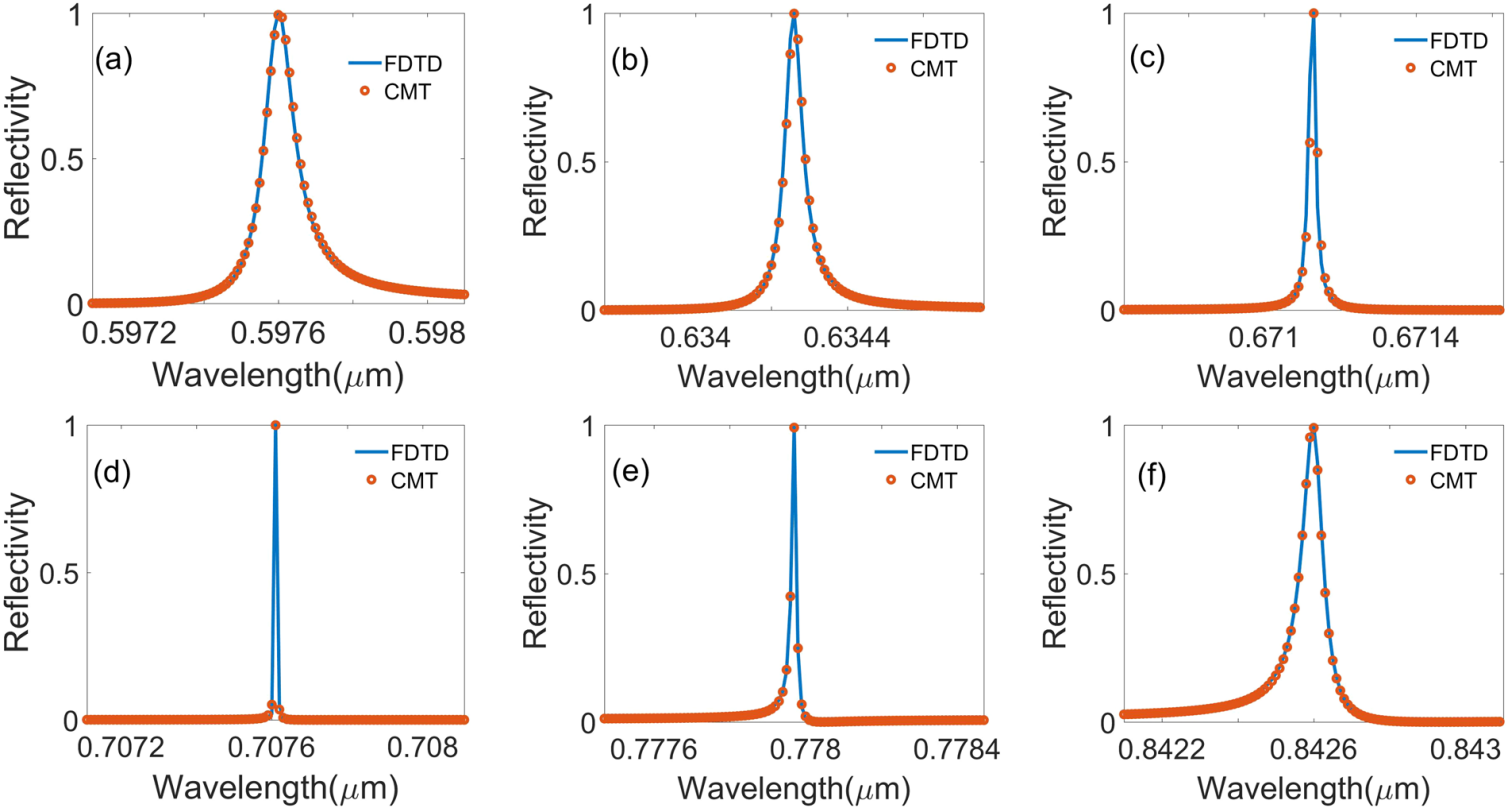
Spectral profiles of zero-order reflectivity of the symmetrical hole array calculated from FDTD (solid lines) and temporal CMT (circle dot) with incident angle (*a*) *θ* = 5 degree, (*b*) *θ* = 10 degree, (*c*) *θ* = 15 degree, (*d*) *θ* = 20 degree, (*e*) *θ* = 30 degree, and (*f*) *θ* = 40 degree, respectively. The parameters *λ*_0_ and *γ* of the theory for the CMT are *λ*_0_ = 604.8581 nm and γ = 2.6e11s^−1^ at *θ* = 5 degree, *λ*_0_ = 617.1255 nm and *γ*  = 1.32e11^−1^ at *θ* = 10 degree, *λ*_0_ = 634.2584 nm and *γ* = 0.46e11 s^−1^ at *θ* = 15 degree, *λ*_0_ = 707.6150 nm and *γ* = 0.08e11 s^−1^ at *θ* = 20 degree, *λ*_0_ = 777.9699 nm and *γ* = 0.22e11 s^−1^ at *θ* = 30 degree, and *λ*_0_ = 842.5980 nm and *γ* = 0.85e11 s^−1^ at *θ* = 40 degree, respectively. Where the slab thickness, annular hole’s inner and outer radii, lattice constant, and surrounding medium refractive index are *h* = 180 nm, *R*_*in*_ = 50 nm, *R*_*out*_ = 100 nm, *a* = 336 nm, and *n* = 1.46, respectively.

In order to improve the results in Fig. 2, we extract the resonance lifetimes from the Fano features in Fig. 3 by describing the guided resonance with FDTD compared with that of the CMT with incident angles θ = 5, 10, 15, 20, 30 and 40 degrees, respectively. The parameters *λ*_0_ and *γ* of the theory for the CMT are *λ*_0_ = 604.8581 nm and *γ* = 2.6e11s^−1^ at *θ* = 5 degree, *λ*_0_ = 617.1255 nm and *γ* = 1.32e11^−1^ at *θ* = 10 degree, *λ*_0_ = 634.2584 nm and *γ*  = 0.46e11 s^−1^ at *θ* = 15 degree, *λ*_0_ = 707.6150 nm and *γ* = 0.08e11 s^−1^ at *θ* = 20 degree, *λ*_0_ = 777.9699 nm and *γ* = 0.22e11 s^−1^ at *θ* = 30 degree, and *λ*_0_ = 842.5980 nm and *γ* = 0.85e11 s^−1^ at *θ* = 40 degree, respectively. Where the slab thickness, annular hole’s inner and outer radii, lattice constant, and surrounding medium index are *h* = 180 nm, *R*_*in*_ = 50 nm, *R*_*out*_ = 100 nm, *a* = 336 nm, and *n* = 1.46, respectively. Figure 3(a–g) show spectral profiles of zero-order reflectivity of the symmetrical hole array calculated from FDTD (solid lines) and temporal CMT (circle dot) for several angles of incidence in a region where BIC occurs. It can be found that all of the resonance peaks have narrow widths. The line width of the reflectivity decreases to zero with the incident angle tending to 23 degree. results from both methods agree each other very well. This phenomenon can be attributed to the coupling loss between the incident mode and the quasi-guide mode being reduced as the incident changes.

**Figure 4:**
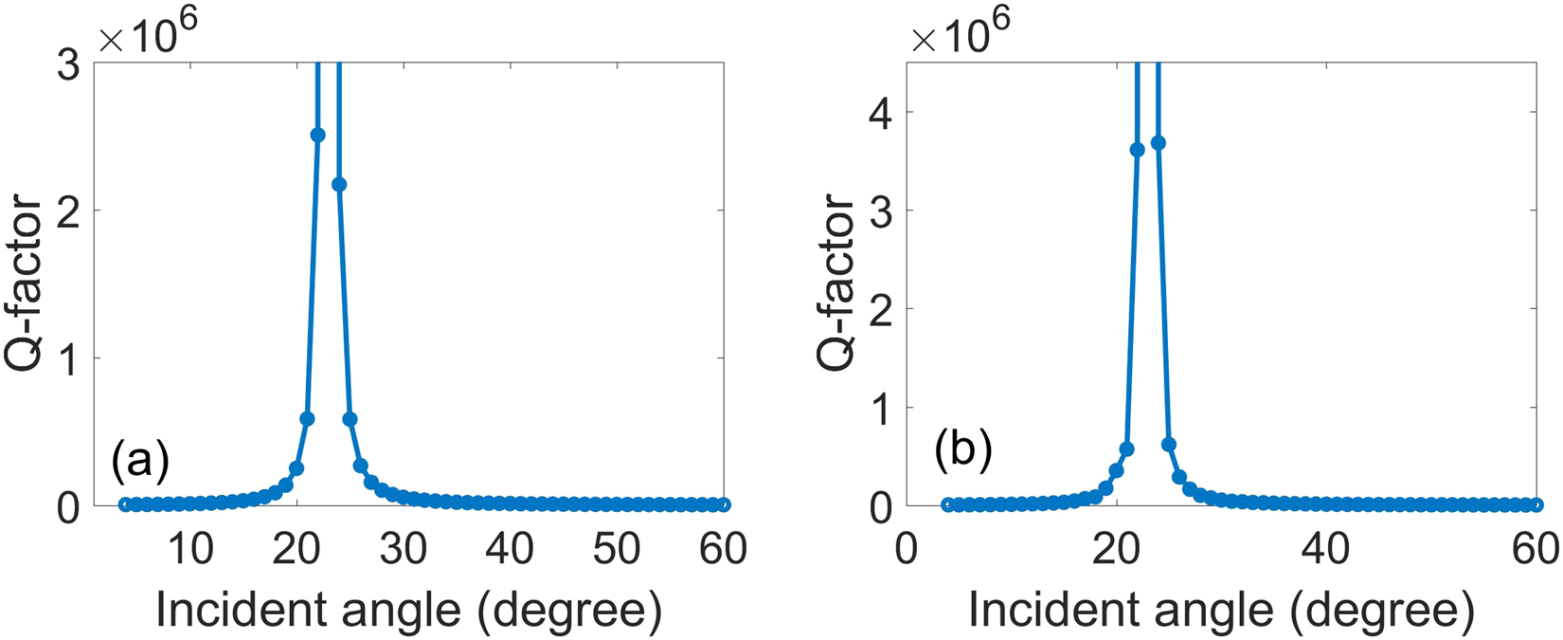
Q-factor of high-Q modes and BICs in the periodic symmetrical annular hole array plotted as a function of incidence angle *θ* from (**a**) the FDTD simulation and (**b**) CMT calculation. Where the slab thickness, annular hole’s inner and outer radii, lattice constant, and surrounding medium refractive index are *h* = 180 nm, *R*_*in*_ = 50 nm, *R*_*out*_ = 100 nm, *a* = 336 nm, and *n* = 1.46, respectively.

Q-factor as a function of incident angle of the high-Q modes in the periodic symmetrical annular hole array, it is defined as *Q* = *Re(g)/2Im(g)*, *g* is the complex eigenfrequency, which has a relationship with the resonance frequency *ω*_0_ and the full width at half maximum (FWHM) of the resonance with *γ* as *g* = *jω*_0_ + *γ/2*. As shown in Fig. 4(a,b), we draw the Q-factor of high-Q modes and BICs in the periodic symmetrical annular hole array as a function of incident angle θ from the FDTD simulation and CMT calculation. It can be obtained that the Q-factor increases significantly between 15 and 30 degrees along the incident angle tending to 23 degree, especially, which can exceed 10^6^ at 23 degree. Results from both methods accord very well. BICs have infinite high Q-factor theoretically, however, it is limited as a result of material absorption, technological intersections, roughness, finite lateral size of samples, and leakage into the substrate in real systems. P mode tends to infinity at an angle of about 23 degree, and the light becomes perfectly confined in the slab, which is the BIC, appearing at a wavelength about 729.17 nm. It means at 23 degree and 729.17 nm, the leaky resonance turns into a localized eigen-mode that does not decay.

**Figure 5:**
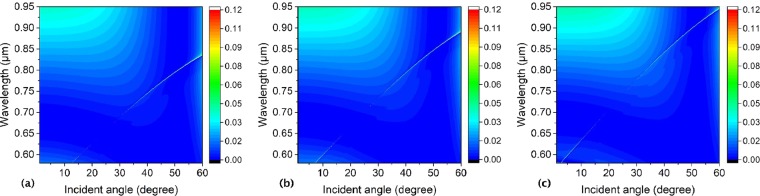
Spectral profiles of zero-order reflectivity of the symmetrical hole array as a function of wavelength *λ* and incident angle *θ* with lattice constant (**a**) *a* = 296 nm, (**b**) *a* = 316 nm, and (**c**) *a* = 336 nm, respectively. Where the slab thickness, annular hole inner and outer radii, and the surrounding medium refractive index are *h* = 180 nm, *R*_*in*_ = 50 nm, *R*_*out*_ = 100 nm, and *n* = 1.46, respectively.

Figure 5 shows spectral profiles of zero-order reflectivity of the symmetrical hole array as a function of wavelength and incident angle with the lattice constant (a) *a* = 296 nm, (b) *a* = 316 nm, and (c) *a* = 336 nm, respectively. Where the thickness of the slab is 180 nm, inner and outer radii are 50 nm, and 100 nm, and the surrounding medium index is 1.46, respectively. As is shown in Fig. 5, all of the reflectivity resonance lines are broken at an incident angle of 23 degree in the three pictures with different lattice constants. However, these reflectivity resonance lines go upper with the lattice constant increasing from 296 nm to 336 nm, which means the center wavelength of the BICs and other Fano resonances redshifts obviously with the lattice constant increasing. The results show that we can obtain BICs at the same incident angle at different wavelengths by tuning the lattice constant of the structure.

**Figure 6:**
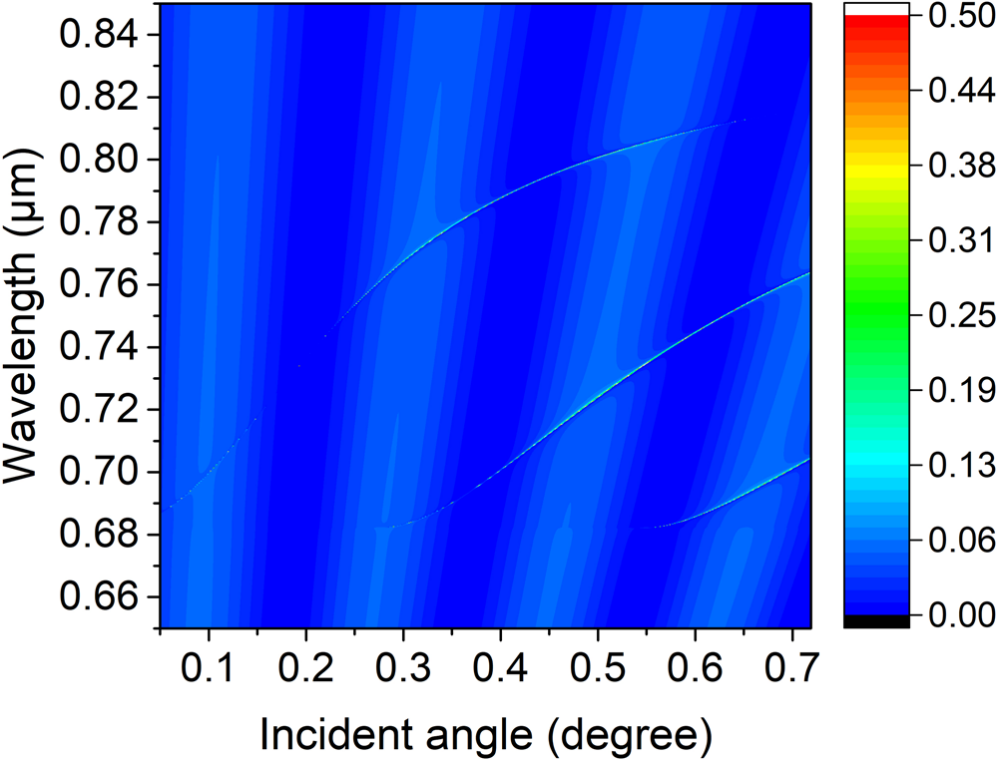
Numerical reflectivity spectra for the p-polarized wave as a function of thickness *h* and wavelength *λ* of the periodic symmetrical annular hole array on a PhC slab. Where the slab annular hole’s inner and outer radii, lattice constant, surrounding medium refractive index and incident angle are *R*_*in*_ = 50 nm, *R*_*out*_ = 100 nm, *a* = 336 nm, *n* = 1.46, and *θ* = 23 degree, respectively.

In order to show the relationship of the PhC slab thickness and BICs, we plot the Fano type reflectivity resonance peak as a function of wavelength *λ* and the PhC thickness *h* in Fig. 6, where slab annular hole’s inner and outer radii, lattice constant, surrounding medium index and incident angle are *R*_*in*_ = 50 nm, *R*_*out*_ = 100 nm, *a* = 336 nm, *n* = 1.46, and *θ* = 23 degree, respectively. As is shown in this picture, three faint reflectivity lines emerge. It can be seen that the top faint Fano resonance line with a high Q-factor is interrupted at a thickness about *h* = 180 nm, which indicates a trapped state with no leakage exists there, and it accords to the results in Fig. 2 well. Additionally, the resonance line vanishes after slab thickness over 650 nm, which means a new BIC there. The middle and the bottom lines are both high Q-factor Fano resonance lines, and BICs take place at left ends of both lines. It means the location and quantity of the BICs can be modulated through slab thickness. Additionally, the sharp Fano resonance peaks redshift obviously with the increase of the slab thickness.

**Figure 7:**
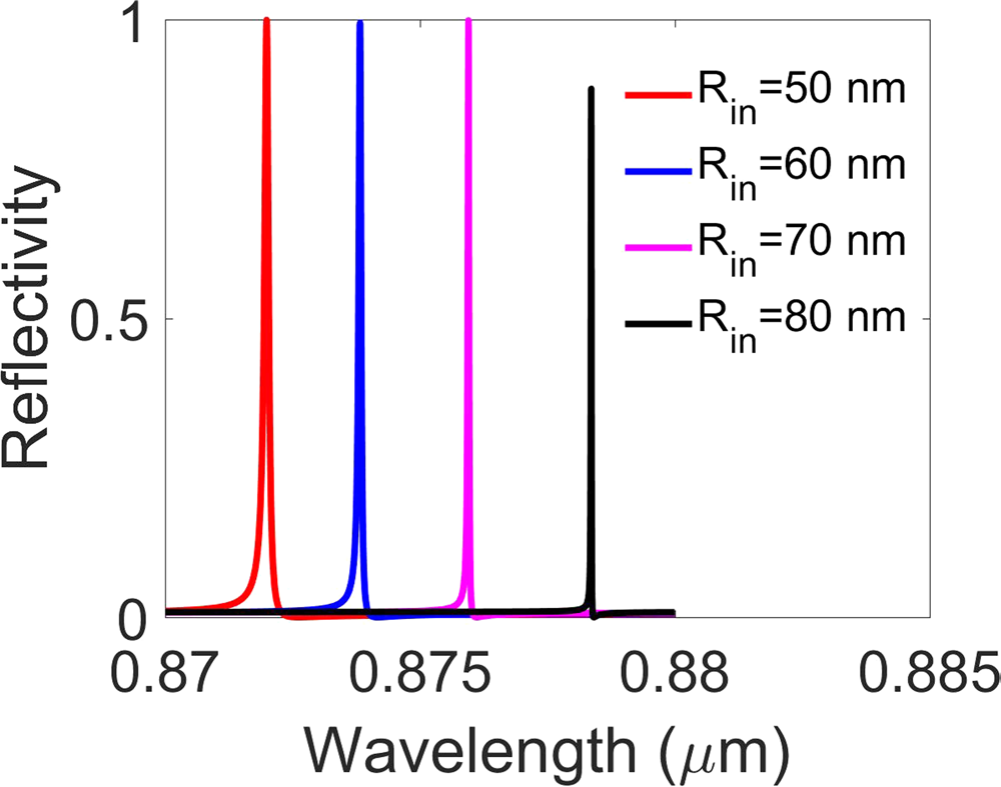
The reflectivity spectra for the p-polarized wave as a function of wavelength *λ* with the annular inner radius being *R*_*in*_ = 50 nm, 60 nm, 70 nm, 80 nm, respectively. Where the slab thickness, annular hole’s outer radii, lattice constant, surrounding medium refractive index and incident angle are *h* = 180 nm, *R*_*out*_ = 100 nm, *a* = 336 nm, *n* = 1.46, and *θ* = 45 degree, respectively.

Additionally, the reflectivity spectra for p-polarized light as a function of wavelength and annular inner radius are simulated as shown in Fig. 7, where the slab thickness, annular hole’s inner and outer radii, lattice constant, surrounding medium index and incident angle are *h* = 180 nm, *R*_*in*_ = 50 nm, *R*_*out*_ = 100 nm, *a* = 336 nm, *n* = 1.46, and *θ* = 45 degree, respectively. It is shown in this figure that the resonance peak redshifts and narrows with the increase of the inner radius at a given incident angle, which means that the resonance line including the BIC can be modulated by the size of the inner radius of the annular holes.

High-Q resonance, especially the infinite Q-factor resonance, in periodic thin films has important potential applications in biochemical sensors, SERS templates, and nonlinear optical components. Q-factor is associated with the field intensity enhancement factor. In order to clarify the BICs, we further investigate the dependence between intensity enhancement factor and the incident angle.

We define two electric field intensity enhancement factors so as to analyze the field enhancement effect. The first one *W*_*V*_ is to evaluate the volume-average intensity inside the PhC slab:

13 $${W}_{V}=\frac{{\int }_{inside}{|{\boldsymbol{E}}/{E}_{0}|}^{2}dV}{{\int }_{inside}dV}$$

**Figure 8:**
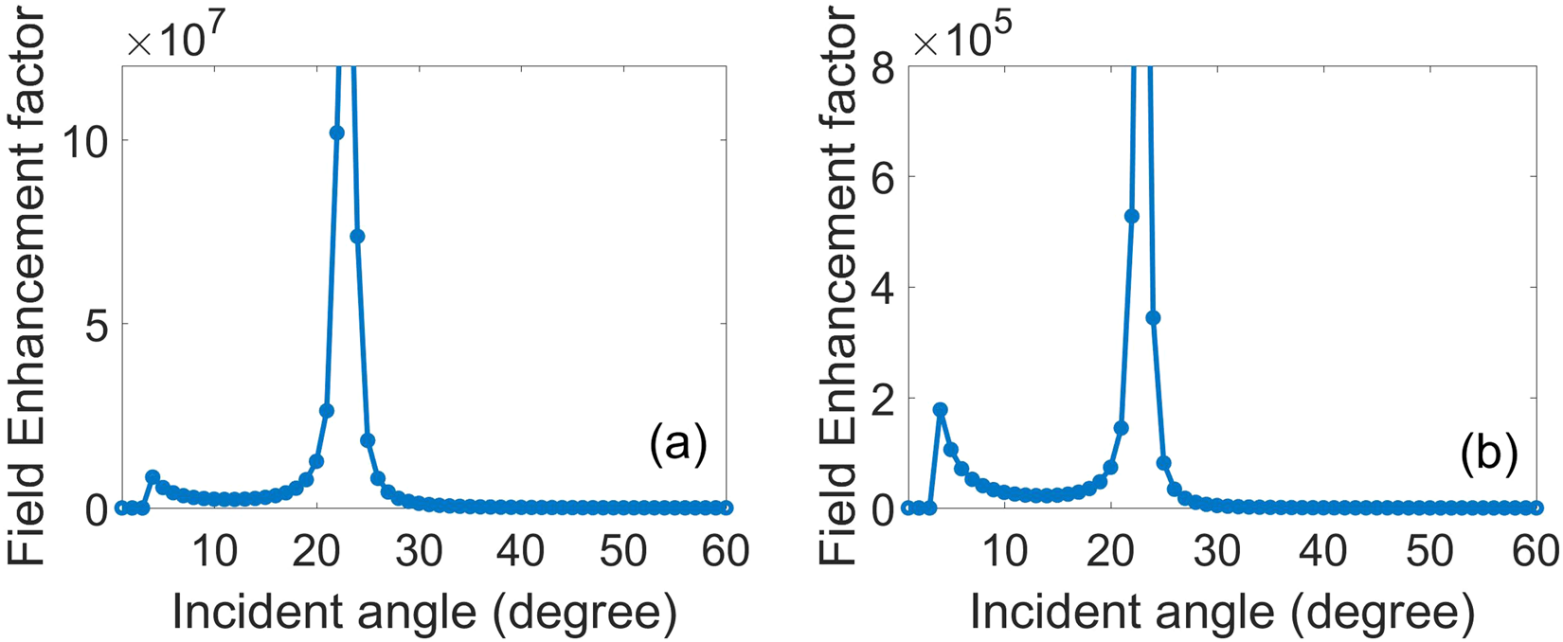
(**a**) Inside electric field intensity enhancement factor *W*_*v*_ and (**b**) Surface electric field intensity enhancement factor *W*_*s*_ of the symmetrical annular hole array. Where the slab thickness, annular hole’s inner and outer radii, lattice constant, and surrounding medium refractive index are *h* = 180 nm, *R*_*in*_ = 50 nm, *R*_*out*_ = 100 nm, *a* = 336 nm, and *n* = 1.46, respectively.

The second one *W*_*s*_ is to calculate the surface-average intensity on the surface of the PhC slab:

14 $${W}_{s}=\frac{{\int }_{surface}{|{\boldsymbol{E}}/{E}_{0}|}^{2}dS}{{\int }_{surface}dS}$$

where ***E*** denotes electric field, *E*_0_ is incident electric field amplitude, *dV* is infinitesimal volume, and *dS* is the infinitesimal surface area. The two enhancement factors *W*_*v*_ and *W*_*s*_ are useful parameters in consideration of a resonance element as a nonlinear optical device using the film’s native optical nonlinearity and SERS template for molecular detection systems, respectively. For resonance Q-factor estimation, the field enhancement factors by the definition in Eqs (13, 14) are directly obtained from the calculated field distributions due to the FDTD. The enhancement factors *W*_*v*_ and *W*_*s*_ in Fig. 8(a,b) show that the electric field intensity is very high, especially, inside the slab and on its surface, it is enhanced by a factor 10^8^, which is highly desirable for new optical devices such as surface emitting amplifiers. The BICs are related to simultaneous suppression of the leakage radiation amplitudes to the zero-order waves in the above and under medium. This effect involves interference between partial leakage radiations from different Bloch modes and complex interaction of evanescent fields at the top and bottom interfaces of the film^15,30^.

**Figure 9:**
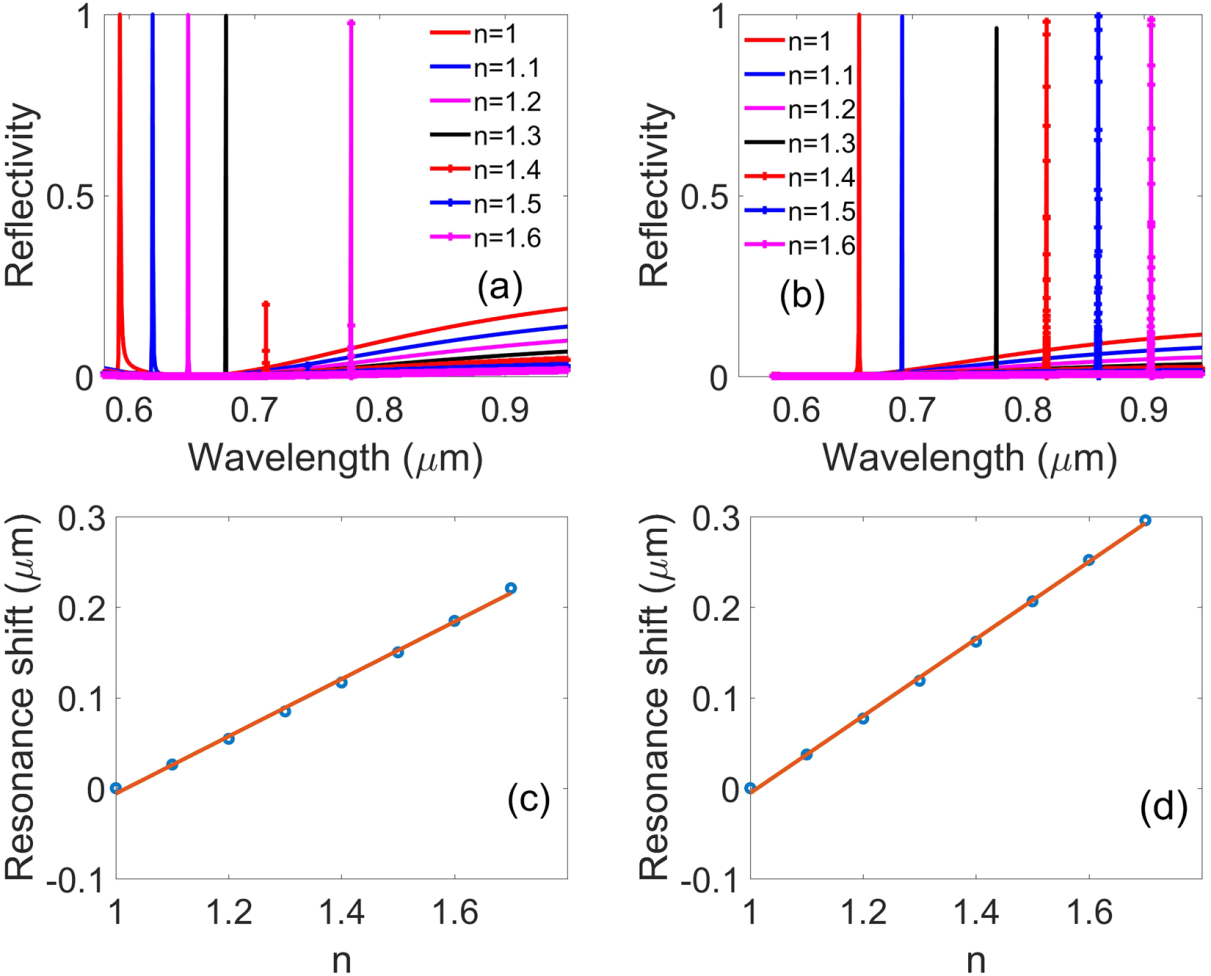
Reflectivity spectra of the symmetrical annular hole array with different surrounding medium with the index from 1 to 1.7 with an incident angle of (**a**) 23 degree and (**b**) 40 degree. Resonance frequency shifts of the symmetrical annular hole array as a function of surrounding medium refractive index *n* with an incident angle of (**c**) 23 degree and (**d**) 40 degree, respectively. Where the slab thickness, annular hole inner and outer radii, and lattice constant, are *h* = 180 nm, *R*_*in*_ = 50 nm, *R*_*out*_ = 100 nm, and *a* = 336 nm, respectively.

For it has very sharp Fano resonance with ultrahigh Q-factor, this annular hole array structure has potential application in sensing. In order to investigate its sensing effect, we simulate the reflectivity spectra at different refractive indexes of the surrounding medium at incident angle 23 degree (Fig. 9(a)) and 40 degree (Fig. 9(b)), where the slab thickness, annular hole inner and outer radii, and lattice constant, are *h* = 180 nm, *R*_*in*_ = 50 nm, *R*_*out*_ = 100 nm, and *a* = 336 nm, respectively. The reflectivity resonance peaks show a significant redshift with the surrounding medium refractive index *n* increasing. It proves that the resonance of the structure is very sensitive to the refractive index of the surrounding medium. In Fig. 9(a), it is shown that the linewidth of the band of the Fano resonance peak in the reflectivity spectra decreases with the surrounding medium refractive index *n* increasing from 1 to 1.4 gradually, then the Fano resonance peak disappears at about *n* = 1.46, which is the BIC as a result of the Fano resonance peak’s bandwidth decreasing to zero. If the refractive index *n* is augmented further, the Fano resonance peak reappears again. Additionally, the decrease of the resonance peak along with the refractive index increasing at about *n* = 1.4 to 1.5 can be attributed to that the bandwidth is too small and it is too hard to catch the highest value in the simulation. Furthermore, when the refractive index is more than 1.7, besides the redshifting resonance peak, a new one comes up around 590 nm, which may be the higher order modes.

**Figure 10:**
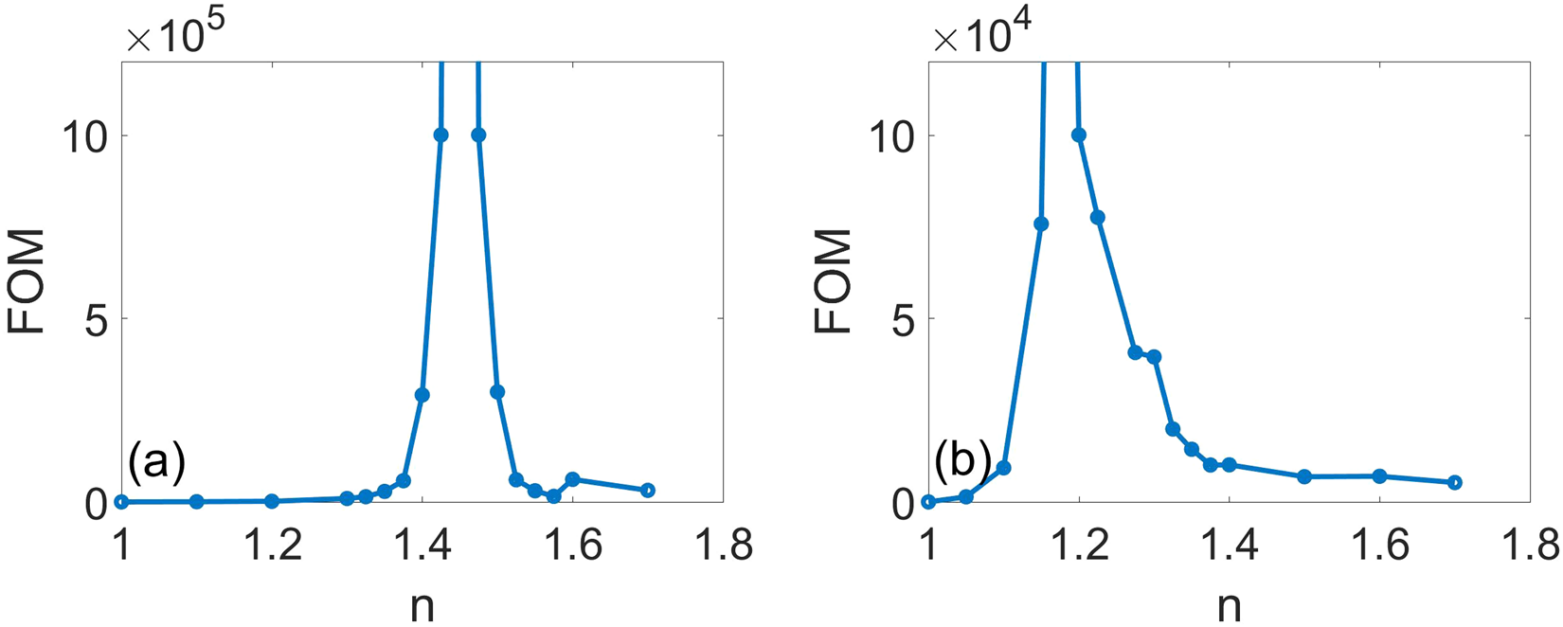
FOM of the symmetrical annular hole array for different surrounding media with refractive index *n* from 1 to 1.7 with an incident angle of (**a**) 23 degree and (**b**) 40 degree. Where the slab thickness, annular hole inner and outer radii, and lattice constant, are *h* = 180 nm, *R*_*in*_ = 50 nm, *R*_*out*_ = 100 nm, and *a* = 336 nm, respectively.

In the figures discussed previously in this paper, it is definitely known if *n* is set as *n* = 1.46, BICs emerge at the incident angle 23 degree, and Fig. 9(a) confirms it once again. We wonder how would the Fano resonance peak behaves with the variation of *n* at an arbitrary incident angle. In this sense, we simulate reflectivity spectra of the structure with an incident angle of 40 degree with different indexes of the surrounding medium in Fig. 9(b). Fano resonance peak in Fig. 9(b) behaves similarly to that in Fig. 9(a). With index *n* increasing, its center resonance wavelength redshifts obviously, and higher order modes are excited around 590 nm when *n* is more than 1.7. What’s more, the resonance peak gets smaller and disappears at *n* = 1.2, and it shows up again as *n* increases continuously after 1.2, which is a BIC. The refractive index of surrounding medium can modulate the location of the tunable BICs which is associated with the changing of the out-of-plane profiles of the individual channels, according to the varying of the surrounding medium refractive index, which modifies the via the continuum coupling weights. In order to illuminate the gradient of the resonance wavelength as a function of the surrounding medium refractive index, we plot a spectral shift vs the refractive index with 23 and 40 degrees in Fig. 9(c,d), and a linear fit to the data reveals that the frequency shift per refractive index unit is about 316.5 nm/RIU and 425.9 nm/RIU, respectively. The figure of merit (FOM) is used for evaluating the performance of a refractive index sensor^31^:

15 $$FOM=\frac{{\rm{\Delta }}{\rm{\lambda }}/{\rm{\Delta }}n({\rm{nm}}/{\rm{RIU}})}{FWHM}$$

where FWHM is the full width at half maximum of the resonance frequency. Here we plot the FOM of the symmetrical annular hole array for a different surrounding media with the index from 1 to 1.7 with an incident angle of Fig. 10(a) 23 degree and Fig. 10(b) 40 degree, respectively. As shown in Fig. 10(a), FOM has a value of more than 10^6^ at the incident angle θ = 23 degree. FOM in Fig. 10(b) is a little lower, but it is still high enough with a value of more than 10^5^ at the incident angle *θ* = 40 degree. The FOM of this annular hole array in the PhC slab is much higher than that of the reported resonances such as Plasmonic induced transmission (PIT), surface plasmon resonance and general Fano resonance. Furthermore, FOM at the BICs can reach infinite theoretically as a result of a zero FWHM, which has potential applications in plasmonic sensors or other nanohole array systems.

**Figure 11:**
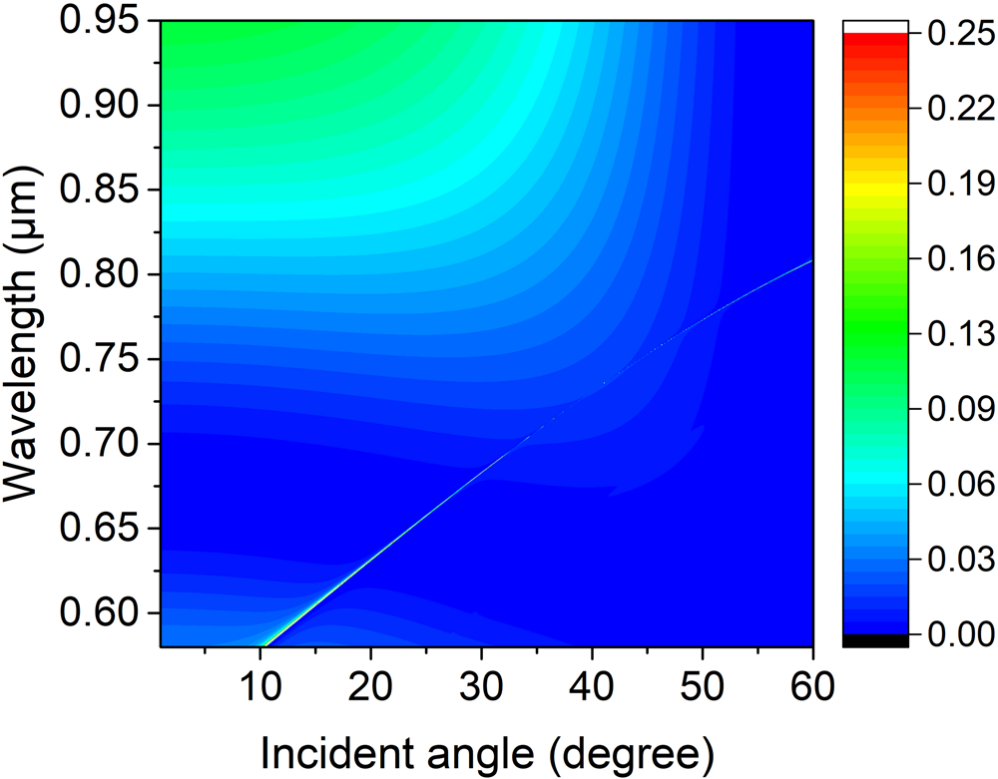
Numerical reflectivity spectra for the p-polarized wave as a function of incident angle *θ* and wavelength *λ* of the periodic symmetrical annular hole array on a PhC slab with a refractive index of the surrounding medium *n* = 1.2. Where the slab thickness, annular hole’s inner and outer radii, lattice constant, and surrounding medium index are *h* = 180 nm, *R*_*in*_ = 50 nm, *R*_*out*_ = 100 nm, *a* = 336 nm, and *n* = 1.2, respectively.

Figure 11 shows the numerical reflectivity spectra for the p-polarized wave as a function of incident angle and wavelength of the periodic symmetrical annular hole array on a PhC Si_3_N_4_ slab with a refractive index of the surrounding medium *n* = 1.2, where the slab thickness, annular hole’s inner and outer radii, lattice constant, and surrounding medium index are *h* = 180 nm, *R*_*in*_ = 50 nm, *R*_*out*_ = 100 nm, *a* = 336 nm, and *n* = 1.2, respectively. A disconnection in the faint reflectivity line is displayed in the map, and light is trapped absolutely there. It means BICs can be acquired at an arbitrary incident angle by modulating the surrounding medium refractive index.

**Figure 12:**
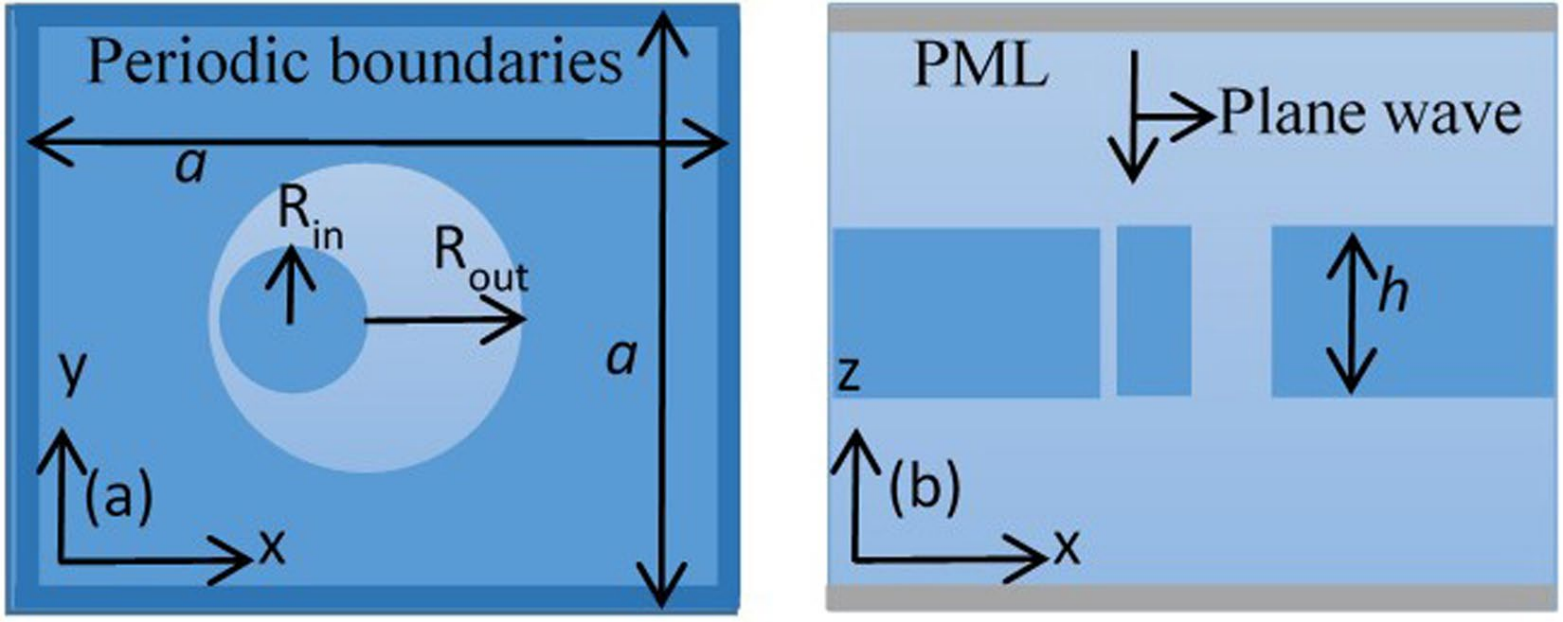
Schematics of Si_3_N_4_ photonic crystal (PhC) slab with square array of asymmetrical annular cylindrical holes. (**a**) Top view of the slab with cylindrical asymmetrical annular hole shapes in *x*-*y* cross-section, and (**b**) side view of the structure in *x*-*z* cross-section.

**Figure 13:**
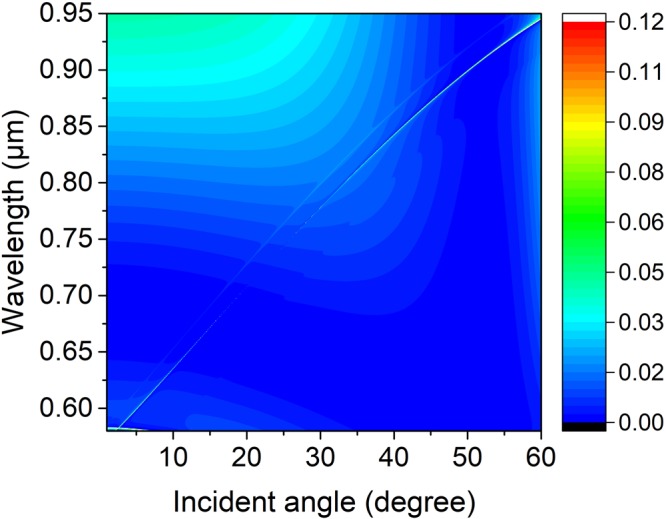
Numerical reflectivity spectra for the p-polarized wave as a function of incident angle and wavelength of the periodic asymmetrical annular hole array on a PhC Si_3_N_4_ slab. Where the slab thickness, annular hole’s inner pillar and outer hole radii, lattice constant, and surrounding medium index are *h* = 180 nm, *R*_*in*_ = 50 nm, *R*_*out*_ = 100 nm, *a* = 336 nm, and *n* = 1.46, displacement between the centers of the inner pillar and the outer hole of the asymmetrical annular hole is *d* = 45 nm, respectively.

All the pictures from Figs 2–11 are plotted in the condition of the hole arrays with symmetrical annular shape in the Si_3_N_4_ PhC slab. In order to investigate the reflectivity property of this structure in all directions, we also consider the annular holes in the slab with an asymmetrical shape as shown in Fig. 12. Figure 12(a) is the top view of the slab with cylindrical asymmetrical annular hole shapes in the *x-y* cross-section and Fig. 12(b) is the side view of the structure in the *x-z* cross-section. Numerical reflectivity spectra for the p-polarized wave as a function of incident angle and wavelength of the periodic asymmetrical annular hole array on a PhC Si_3_N_4_ slab is plotted in Fig. 13, where the slab thickness, annular hole’s inner and outer radii, lattice constant, and surrounding medium index are *h* = 180 nm, *R*_*in*_ = 50 nm, *R*_*out*_ = 100 nm, *a* = 336 nm, and *n* = 1.46, respectively, and displacement between the centers of the inner pillar and the outer hole of the asymmetrical annular hole is *d* = 45 nm. The reflectivity line in Fig. 13 shows up a faint reflectivity line, and it is disconnected at around incident angle of 23 degree, which means there is a BIC in the structure with an asymmetrical annular shape. Compared with that in Fig. 2, a new fainter reflectivity line parallel to the original one emerges, which means a new sharper mode as a form of Fano resonance comes up as a result of the modes coupling associated with the asymmetrical hole shape. Furtherly, it disappears at both ends of the new reflectivity line, which may also be attributed to new BICs.

**Figure 14:**
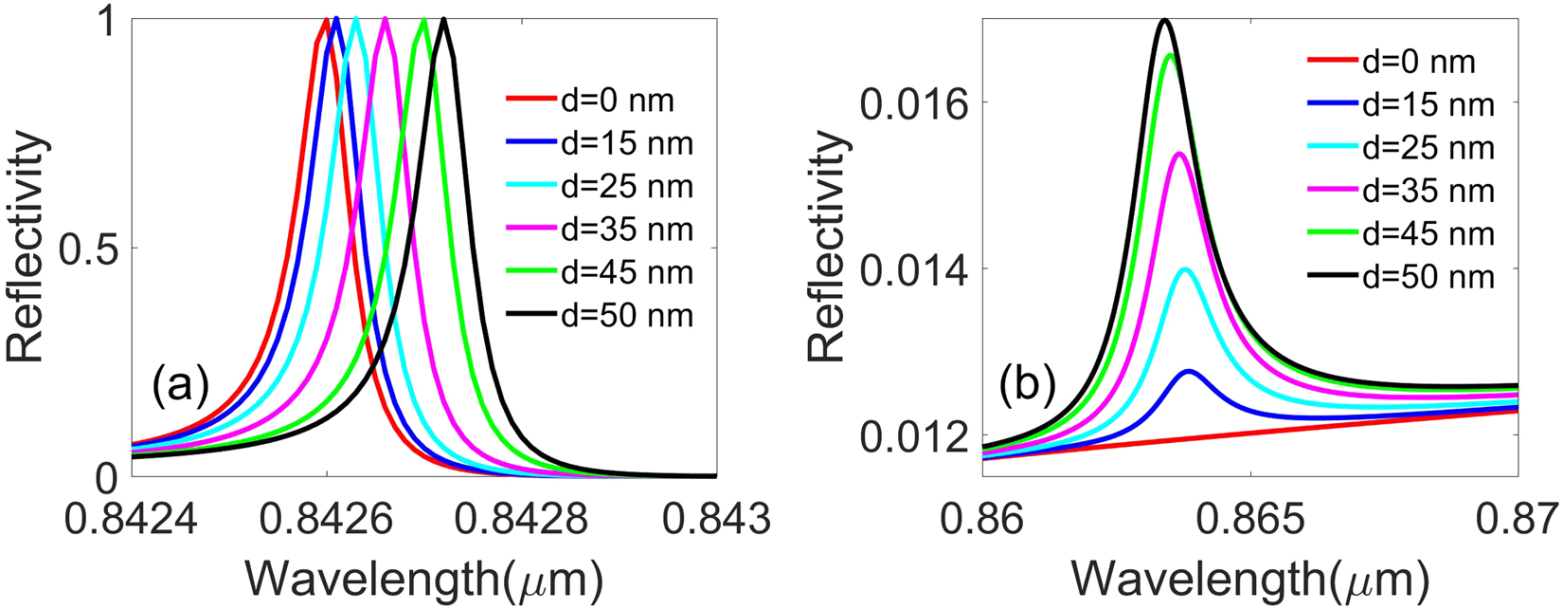
The reflectivity spectra for the p-polarized wave as a function of wavelength with the displacement between the centers of the inner pillar and the hole radii being *d* = 0, 15 nm, 25 nm, 35 nm, 45 nm and 50 nm, respectively. (**a**) The original resonance of the annular hole array, (**b**) The new resonance as a result of the asymmetrical shape of the annular hole array. Where the slab thickness, annular hole’s inner pillar and outer hole radii, lattice constant, surrounding medium index, and the incident angle are *h* = 180 nm, *R*_*in*_ = 50 nm, *R*_*out*_ = 100 nm, *a* = 336 nm, *n* = 1.46, and *θ* = 40 degree, respectively.

We also plot the reflectivity spectra for p-polarized wave as a function of wavelength with the displacement between the centers of the inner pillar and outer holes being *d* = 0 nm, 15 nm, 25 nm, 35 nm, 45 nm, and 50 nm, respectively, as shown in Fig. 14, where the slab thickness radii of the annular hole’s inner pillar and outer hole, lattice constant, surrounding medium refractive index, and the incident angle are *h* = 180 nm, *R*_*in*_ = 50 nm, *R*_*out*_ = 100 nm, *a* = 336 nm, *n* = 1.46, and *θ* = 40 degree, respectively. Figure 14(a) shows the original reflectivity resonance of the annular hole array. It is obtained that the reflectivity resonance line redshifts regularly with the displacement between the centers of the inner pillar and the outer hole increasing from 0 to 50 nm, moreover, a new reflectivity resonance peak appears at around 0.864 μm. Additionally, with the displacement increasing, we can see the new reflectivity peak gets higher and wider, but the center wavelength of the peak stays at the same place, as shown in Fig. 14(b). The new reflectivity resonance of the asymmetrical annular hole array may be associated with the mode coupling between the inner pillar and the PhC slab. From Fig. 14, we infer that the BICs in this annular hole array can be modulated by the displacement of the inner pillar to the hole center.

**Figure 15:**
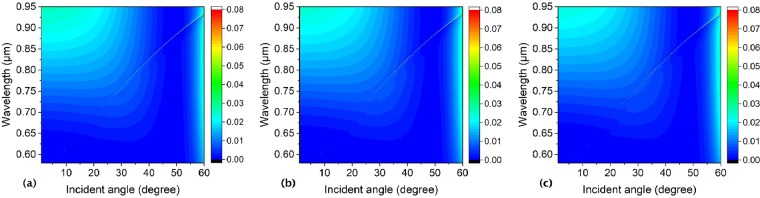
Numerical reflectivity spectra for the p-polarized wave as a function of incident angle and wavelength of the periodic annular hole array on a PhC Si_3_N_4_ slab with two identical inner pillars moving to each other inside the outer hole at symmetrical locations. The center of the outer hole is (*x*, *y*) = (0, 0), and centers of the two inner pillars are (**a**) (−0.095 μm, 0), (0.095 μm, 0), (**b**) (−0.075 μm, 0), (0.075 μm, 0), and (**c**) (−0.055 μm, 0), (0.055 μm, 0). Where the slab thickness, annular hole’s inner pillars and outer hole radii, lattice constant, and surrounding medium index are *h* = 180 nm, *R*_*in1*_ = *R*_*in2*_ = 50 nm, *R*_*out*_ = 150 nm, *a* = 336 nm, and *n* = 1.46, respectively.

In order to furtherly prove the impact of the asymmetry on the BICs formation, we explore the reflectivity of the annular hole array with two inner pillars inside each hole. Firstly, the reflectivity is studied of the hole array with two identical inner pillars at symmetric locations and move to each other regularly with the centers of the outer hole and the two inner pillars in a line. The outer hole center is (*x*, *y*) = (0, 0), and centers of the two inner pillars move to each other regularly from (a) (−0.095 μm, 0), (0.095 μm, 0), to (b) (−0.075 μm, 0), (0.075 μm, 0), and to (c) (−0.055 μm, 0) then to (d) (0.055 μm, 0). As is shown in Fig. 15, we can find only one reflectivity line, and the reflectivity line with the BICs in it changes very insignificantly with the two inner pillars moving to each other at symmetrical locations. Where the slab thickness, annular hole’s inner pillars and outer hole radii, lattice constant, and surrounding medium index are *h* = 180 nm, *R*_*in1*_ = *R*_*in*2_ = 50 nm, *R*_*out*_ = 150 nm, *a* = 336 nm, and *n* = 1.46, respectively.

**Figure 16:**
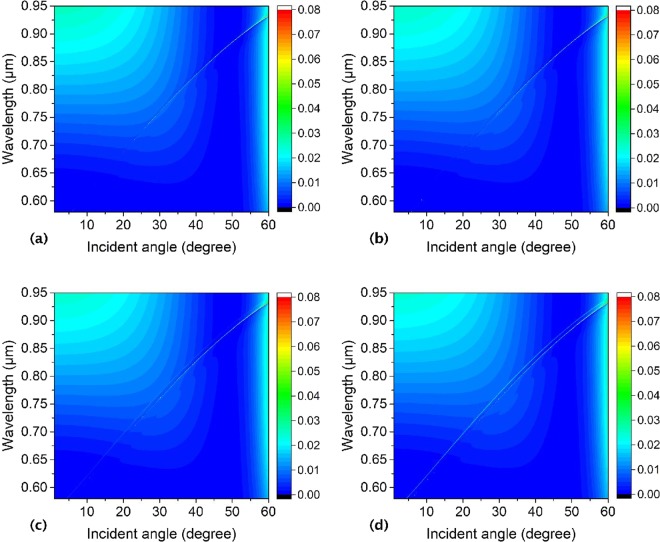
Numerical reflectivity spectra for the p-polarized wave as a function of incident angle and wavelength of the periodic annular hole array on a PhC Si_3_N_4_ slab with two identical inner pillars inside the outer hole array, one staying and the other one moving to it. The center of the outer hole is (*x*, *y*) = (0, 0), and centers of the two inner pillars are (**a**) (−0.095 μm, 0), (0.095 μm, 0), (**b**) (−0.095 μm, 0), (0.075 μm, 0), (**c**) (−0.095 μm, 0), (0.055 μm, 0) and (**d**) (−0.095 μm, 0), (0.025 μm, 0). Where the slab thickness, annular hole’s inner pillars and outer hole radii, lattice constant, and surrounding medium index are *h* = 180 nm, *R*_*in1*_ = *R*_*in2*_ = 50 nm, *R*_*out*_ = 150 nm, *a* = 336 nm, and *n* = 1.46, respectively.

We also investigate the reflectivity of the hole array with two identical inner pillars inside the outer hole with one inner pillar staying at (−0.095 μm, 0) and the other one moving to it from a symmetrical location (0.095 μm, 0) to an asymmetrical one, and the centers of the outer hole and the two inner pillars are in a line. It shows only one reflectivity line when the other pillar at (0.095 μm, 0) in Fig. 16(a). However, as this pillar moves to the first one from (0.095μm, 0) to (0.025 μm, 0), a new reflectivity resonance line appears, and it is more and more obvious as shown in Fig. 16(b–d) as a result of symmetry broken of the annular holes. If the location of the moving inner pillar is tuning suitably a new BIC with zerowidth in the new reflectivity Fano resonance line can be obtained. The slab thickness, annular hole’s inner pillars and outer hole radii, lattice constant, and surrounding medium index of this structure are *h* = 180 nm, *R*_*in1*_ = *R*_*in*2_ = 50 nm, *R*_*out*_ = 150 nm, *a* = 336 nm, and *n* = 1.46, respectively.

**Figure 17:**
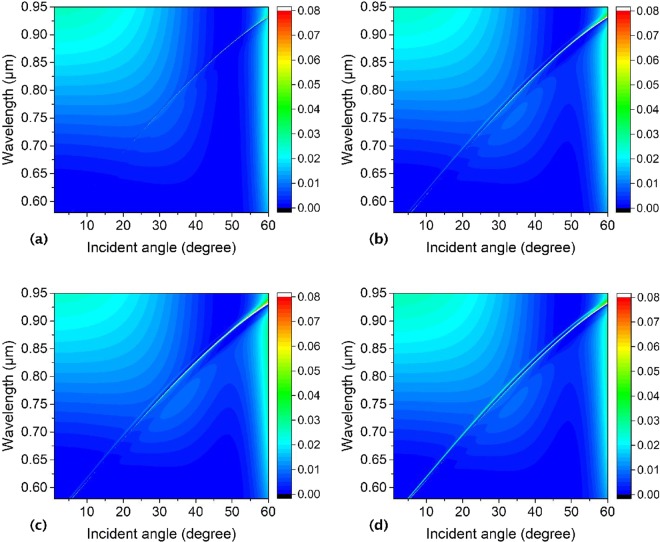
Numerical reflectivity spectra for the p-polarized wave as a function of incident angle and wavelength of the periodic asymmetrical annular hole array on a PhC Si_3_N_4_ slab with two identical inner pillars inside the outer hole array, one staying and the other one moving around the outer hole. The center of the outer hole is (*x*, *y*) = (0, 0), and centers of the two inner pillars are (**a**) (−0.095 μm, 0), (0.095 μm, 0), (**b**) (−0.095 μm, 0), (0.0672 μm, 0.0672 μm), (**c**) (−0.095 μm, 0), (0, 0.095 μm) and (**d**) (−0.095 μm, 0), (0.0672 μm, −0.0672 μm). Where the slab thickness, annular hole’s inner pillars and outer hole radii, lattice constant, and surrounding medium index are *h* = 180 nm, *R*_*in1*_ = *R*_*in2*_ = 50 nm, *R*_*out*_ = 150 nm, *a* = 336 nm, and *n* = 1.46, respectively.

Furthermore, the reflectivity is studied of the hole array with two identical inner pillars inside the outer hole with one inner pillar staying and the other one moving around the outer hole. If the two inner pillars are arranged symmetrically inside each hole with centres at (−0.095 μm, 0) and (0.095 μm, 0), only one reflectivity line shows up as shown in Fig. 17(a). When one of the two inner pillars stays at (−0.095 μm, 0), with the other one moving from (0.095 μm, 0) to (0.0672 μm, 0.0672 μm), then to (0, 0.095 μm), at last to (−0.0672 μm, 0.0672 μm), new reflectivity lines aside the original one will turn up (as shown in Fig. 17(b–d)), which are also Fano resonances. Fano resonances originated from the coupling between the guided modes supported by the slab and external plane waves. With the two inner pillars’ location symmetry being broken, the dispersion relationship is altered, and the waveguide modes and coupling between them are changed, so new Fano resonance lines show up, and its width can be modulated by the inner pillars’ locations. If the Fano resonance line is faint to zerowidth, a new BIC is formed. Where the slab thickness, annular hole’s inner pillars and outer hole radii, lattice constant, and surrounding medium index are *h* = 180 nm, *R*_*in1*_ = *R*_*in2*_ = 50 nm, *R*_*out*_ = 150 nm, *a* = 336 nm, and *n* = 1.46, respectively.

We discuss the reflectivity of the annular hole arrays with two identical inner pillars inside each hole with symmetrical and asymmetrical locations, and it is found that if the two inner pillars inside the hole have symmetrical locations, there is only one reflectivity line with BICs. However, if the location symmetry is broken, new reflectivity line and BICs appear. This means the location symmetry of the two identical inner pillar can modulate the reflectivity properties of the whole structure.

**Figure 18:**
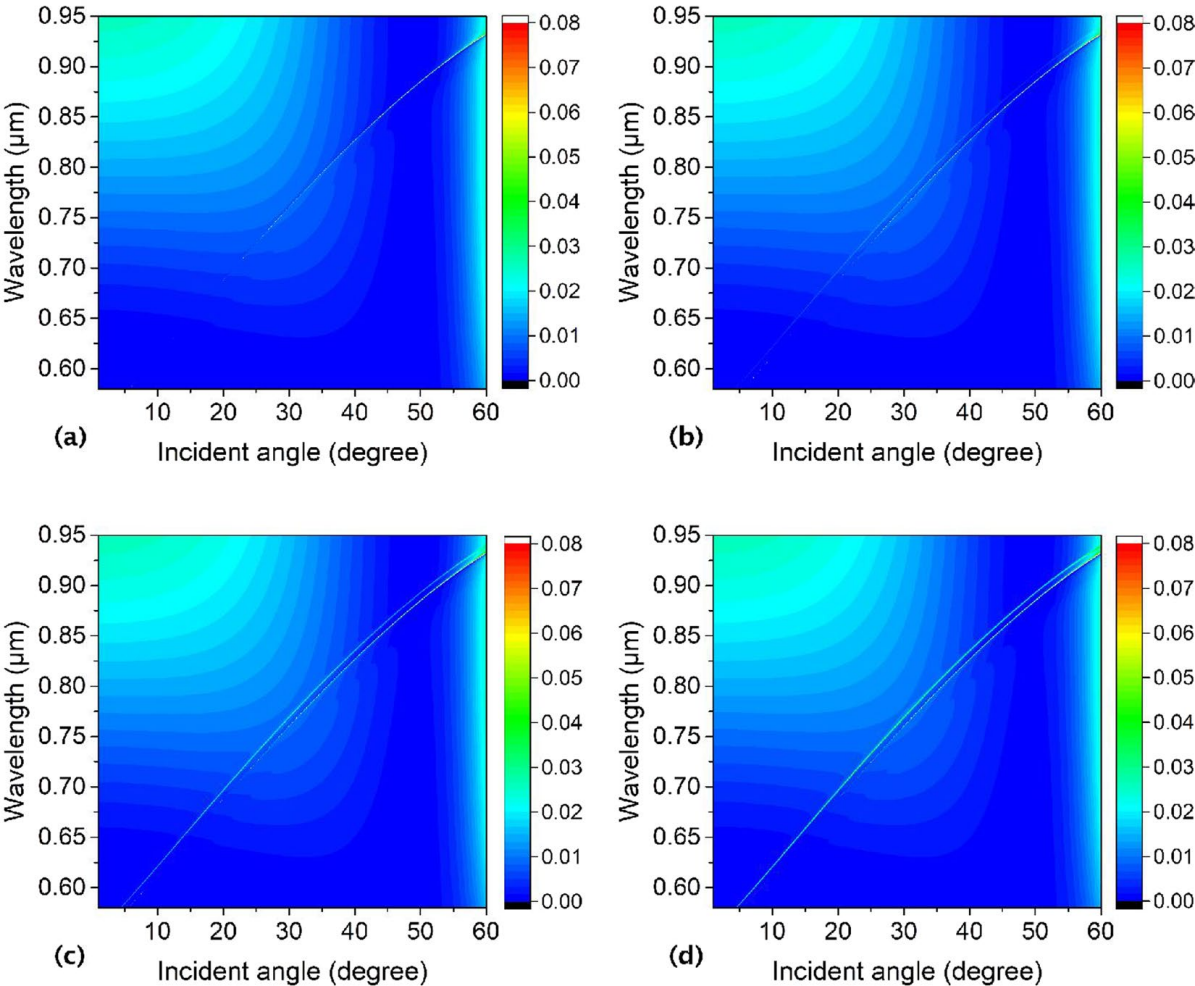
Numerical reflectivity spectra for the p-polarized wave as a function of incident angle and wavelength of the periodic asymmetrical annular hole array on a PhC Si_3_N_4_ slab with two inner pillars inside the outer holes with different radii (**a**) *R*_*in1*_ = 0.05 μm, *R*_*in2*_ = 0.05 μm with centers at (−0.095 μm, 0), (0.095 μm, 0), (**b**) *R*_*in1*_ = 0.04 μm, *R*_*in2*_ = 0.0583 μm with centers at (−0.105 μm, 0), (0.0867 μm, 0), (**c**) *R*_*in1*_ = 0.03 μm, *R*_*in2*_ = 0.064 μm with centers at (−0.115 μm, 0), (0.081 μm, 0), and (**d**) *R*_*in1*_ = 0.02 μm, *R*_*in2*_ = 0.0678 μm with centers at (−0.125 μm, 0), (0.0772 μm, 0). The center of the outer hole is (*x*, *y*) = (0, 0), and it is in a line with the centers of the two inner pillars. Where the slab thickness, annular hole’s outer hole radii, lattice constant, and surrounding medium index are *h* = 180 nm, *R*_*out*_ = 150 nm, *a* = 336 nm, and *n* = 1.46, respectively.

At last, we will talk over the reflectivity of the annular hole array with two inner pillars inside each hole with different sizes. As is shown in Fig. 18(a), only one reflectivity line with BICs shows up when the two inner pillars with the same size at symmetrical locations. Along with the size difference amplifying between the two inner pillars, new Fano resonance line emerges and it can be modulated regularly (as shown in Fig. 18(b–d)), and new BIC can show up with the radii of the two inner pillars tuning finely, which is associated with the coupling between the changed guided modes supported by the slab and external plane waves. Additionally, the radii of the two inner pillars are set as *πR*_*in1*_^*2*^ + *πR*_*in2*_^*2*^ = *π*(0.05 μm)^2^ + *π*(0.05 μm)^2^. The center of the outer hole is (*x*, *y*) = (0, 0). Where the slab thickness, annular hole’s outer hole radii, lattice constant, and surrounding medium index are *h* = 180 nm, *R*_*out*_ = 150 nm, *a* = 336 nm, and *n* = 1.46, respectively.

The results above mean the location asymmetry and size asymmetry of the inner pillars inside the annular hole array can impact the reflectivity and the formation of the BICs obviously.

The BICs discussed in this paper are ones at the nonzero incident angle, which are the tunable off –Γ BICs due to the weighted destructive via the continuum interference in the vicinity of accidental symmetry when the majority of the radiation is precanceled. Unlike the stationary at-Γ BICs originated from the geometry symmetry of the structure, the tunable off-Γ BICs in the PhC slabs on TM-like bands^15^ at some seemingly unremarkable wave vectors without symmetry incompatibility, giving rise to a tunable trapping of light^28^.

In the formation of tunable BICs in TM-like modes, the surface coupling plays an important role. The surface coupling precompensates the majority of the large leakage caused by the in-plane coupling^28^. The formation of the tunable BICs is the partial cancellation of the in-plane and the surface coupling ensures rather low radiation ability of the separate closed channels. New accidental symmetry induces strong coupling in via the continuum channels. Finally, scanning of the wave vector allows achieving the weighted destructive interference between the closed and open channels.

In this paper, we prove that the tunable BICs of the annular hole array show up when the holes are symmetry. When the shape symmetry of one lattice of annular hole array is broken, there appears a new Fano resonance reflectivity line accompanying the original one. It is known that the Fano resonances originated from the coupling between the guided modes supported by the slab and external plane waves. With the symmetry of the annular hole being broken, the dispersion relationship is altered, and the waveguide modes and coupling between them are changed. As a result, new Fano resonance line will emerge and the line width of it can be effectively modulated by tuning the inner pillars’ location and size symmetry of the annular hole. When the linewidth of Fano resonances tends to zero, the light is perfectly confined in the slab, and BICs can be observed in the Fano resonance lines at nonzero incident angles.

## Conclusion

In conclusion, it is shown that, for a Si_3_N_4_ PhC slab with periodic annular hole array immersed in the silica medium, BICs with a zerowidth can be found in the reflectivity spectra. It shows that this structure supports extremely strong field enhancement with a factor more than 10^8^ associated with the BICs. Light can be perfectly confined in this annular hole array at suitable lattice constant, slab thickness, annular hole outer and inner radii, and medium refractive indexes surrounding the PhC slab. FOM at the BICs can reach infinite theoretically as a result of a zero FWHM. Moreover, New Fano resonance line appears with BICs when the annular hole’s symmetry is broken, which may be attributed to the change of the waveguide modes and their coupling when the annular hole shape is asymmetrical. We confirm it by tuning the inner pillars’ location and size to realize the structure’s asymmetry. It is shown the location and size asymmetry of the inner pillars inside each outer hole can impact the reflectivity and the formation of the BICs obviously. In order to prove the validity of the investigation, we compare the FDTD simulation results with that of the CMT calculations. Results from both methods agree very well. Results in this paper are helpful in the design of practical resonance elements based on optical BICs in various applications, such as biosensors, perfect filters, and waveguides.
